# Normal and altered masticatory load impact on the range of craniofacial shape variation: An analysis of pre-Hispanic and modern populations of the American Southern Cone

**DOI:** 10.1371/journal.pone.0225369

**Published:** 2019-12-11

**Authors:** Andrea P. Eyquem, Susan C. Kuzminsky, José Aguilera, Williams Astudillo, Viviana Toro-Ibacache

**Affiliations:** 1 Centro de Análisis Cuantitativo en Antropología Dental, Facultad de Odontología, Universidad de Chile, Santiago, Chile; 2 Max Planck Weizmann Center for Integrative Archaeology and Anthropology, Max Planck Institute for Evolutionary Anthropology, Leipzig, Germany; 3 Department of Anthropology and Applied Archaeology, Eastern New Mexico University, Portales, New Mexico, United States of America; 4 Anthropology Department, University of California, Santa Cruz, California, United States of America; 5 Facultad de Medicina and Hospital Clínico, Universidad de Chile, Santiago, Chile; 6 Department of Human Evolution, Max Planck Institute for Evolutionary Anthropology, Leipzig, Germany; The Cyprus Institute, CYPRUS

## Abstract

The reduction of masticatory load intensity resulting from dietary changes in human evolution has been proposed as an important factor that alters craniofacial shape in past and current populations. However, its impact on craniofacial variation and on the perceived differences among populations is unclear. The maxillomandibular relationship, which alters masticatory force direction, is a factor often neglected but it can contribute to variation in craniofacial morphology, particularly among modern/urban populations where the prevalence of dental malocclusions is greater than in prehistoric populations. This study investigates the influence of masticatory load intensity and maxillomandibular relationship as a proxy for force direction on the human craniofacial skeleton. By using 3D imaging and geometric morphometrics, we analyzed craniofacial shape variation among 186 individuals from pre-Hispanic and modern Chilean and Argentinean populations that differ in diet consistency (a proxy for masticatory load intensity) and maxillomandibular relationship. We predicted that masticatory load would have a subtle effect on the upper craniofacial bones and that this would be more marked in the maxilla. Our results showed no clear influence of masticatory load on craniofacial shape, particularly in modern/urban populations. Allometry, on the contrary, shows a stronger effect. The degree of integration between the upper craniofacial bones and the load-bearing maxilla depends on masticatory load intensity, decreasing from high to low but showing a conservative pattern of covariation among the groups. The degree of variation in the shape of the maxilla is greater than the upper craniofacial bones. These results suggest that masticatory load has a limited effect in determining differences in craniofacial morphology among populations. This effect is slightly greater for the maxillary region of the face. We propose that the reduction of functional constraints is key to greater shape variation found in modern/urban populations.

## Introduction

The assessment of human skull shape has been key for researchers reconstructing prehistoric population history [1–5]. These studies rely on the assumption that skull shape is primarily controlled by neutral evolutionary factors [6]. However, a complicating factor is adaptation to climate, which may also influence skull shape in populations [7–9]. In addition, pathologies of the hard tissues and other plastic changes that can affect bone due to physiological factors may alter skull shape [10, 11]. One such example is the presence of dental malocclusion, which tends to be more frequent in modern/urban populations who eat a heavily processed diet. While some researchers have addressed underlying epidemiological changes (e.g., [11, 12]), most anthropological research has proposed that malocclusions may have appeared as a consequence of dietary changes [11, 13, 14, 15]. Yet, there is a dearth of research that addresses the effect that such malocclusions have on skull shape variation among populations. Some researchers report that a high prevalence of different dental malocclusions in modern populations is an important aspect of variation in the modern human skull [see 16, 17, 18, 19 for examples]. In a study of Swedish populations, approximately 52.3% of occlusal anomalies have been reported [20]. In a sample of Colombian children and adolescents from Bogotá, dental anomalies and/or malocclusions were found in more than three-quarters of the sampled population, while 57.3% of a sample of Brazilian adults also have dental malocclusions [21, 22]. Some evidence suggests that people living in medieval Europe also had dental malocclusions [23]. Historical samples from Croatia show slight temporal differences in their prevalence of dental malocclusions, with lower rates reported during the 3^rd^-5^th^ centuries, which seems to have increased later in the 6^th^-10^th^ centuries [24]. Among dental malocclusions, alterations of the maxillomandibular relationship leads to changes in the relative position of muscles, the temporomandibular joint (TMJ) and teeth and thus, force directions [25]. The high prevalence of dental anomalies in modern groups, particularly those affecting the relationship between the maxilla and the mandible, makes them an interesting, yet largely unexplored model to investigate the extent to which different aspects of masticatory loads, such as force magnitude and maxillomandibular relationship (as a proxy for force direction) cause variation among populations.

Plastic changes due to bone remodeling in response to loads are relevant in shaping the skull in mammals [26–28]. It is then plausible that craniofacial variation may be attributed to developmental trajectories that have been affected by how bones have been loaded throughout life. From a biomechanical perspective, chewing is the most important source of loading acting on the skull [29, 30].

Variation of masticatory force magnitudes and their effect on craniofacial shape variation could reflect shifts in subsistence economies. In the case of dental malocclusions, these also have a genetic origin [31–33], but they can be amplified by the resulting altered mechanical loading [32]. In both prehistoric and modern populations there has been a shift from hunter/gathering/fishing lifeways to agriculture and in more recent contexts, the reliance on highly processed food items prevalent in the western diet [13, 14]. The nutritional content of such diets (protein, minerals, fiber, carbohydrates) is also different and can affect basal metabolism and bone development [11, 34]. Mechanically, the more food is pre-processed, the lower the masticatory effort required to break it down and the lower the forces experienced by the facial bones [30, 35, 36]. In general terms, reduced strain on bones would result in a reduction in bone growth and this has been proposed as a relevant factor in skull variation among populations, particularly in the lower portion of the face, with the rest of the bones being subject to the influence of other agents of variation [37, 38]. In the case of modern individuals, who eat a soft, mechanically less demanding diet, mandibular prognathism would result from an excessive growth of the condyle cartilage; this has been proposed to be a response to masticatory loads of an individual’s mandible, the interaction between genes and environmental factors, which can increase the predisposition to develop such malocclusions [32].

Studies addressing the topic of dietary changes and skull shape have reached somewhat different conclusions. Galland, van Gerven [39] showed that hunter-gatherers had wider and shorter faces, wider zygomatic bones, more pronounced alveolar prognathism, lower and wider orbits and smaller nasal aperture when compared with agriculturalists. In contrast, Paschetta, de Azevedo [40] found that the agricultural transition was not necessarily associated with a reduction in overall robusticity or size. Instead, the authors reported that hunter-gatherers had relatively smaller structures than agriculturalists with subtle morphological differences; the larger differences were found in the position of the superior attachment of the temporalis muscle, anterosuperiorly positioned in hunter-gatherers. von Cramon‐Taubadel [14] found that the mandible is the structure most affected by masticatory forces, followed by–and less influenced by–the palatomaxilla region, which is also closely integrated with the mandible; while the upper face and vault would reflect more the genetic population history of the individuals. Toro-Ibacache, Ugarte [19], using part of the sample included in the present study, showed that the effect of masticatory load intensity and maxillomandibular relationship on mandible shape, and the inner amount of cortical bone and mechanical resistance to deformation is limited. Further, the authors found that, a more marked allometric effect that might be linked to other variables, such as nutrition or basal metabolism. Because there is a general consensus that masticatory load affects the shape of the mandible, and to a greater extent than the craniofacial skeleton [19, 38, 41, 42], the focus of this work is centered on the latter, which is one of the structures most widely used in anthropology for the study of the origin and life history of human populations. This is also relevant in dentistry to aid in elucidating the extent to which the cranium, a structure with a strong developmental constraint linked to its many functions, participates in the alterations of maxillomandibular relationships.

The aim of this study is to examine the intensity of masticatory load, and how the altered direction of forces occurring during altered maxillomandibular relationships, plays a role in shape variation of the human skull. We test the hypothesis that there is a relationship between masticatory load intensity, maxillomandibular relationship and craniofacial shape. Our prediction is that both masticatory load intensity and maxillomandibular relationship show only a subtle association with overall craniofacial shape, and that the association is stronger in the maxilla in both cases.

## Materials and methods

**Fig 1:**
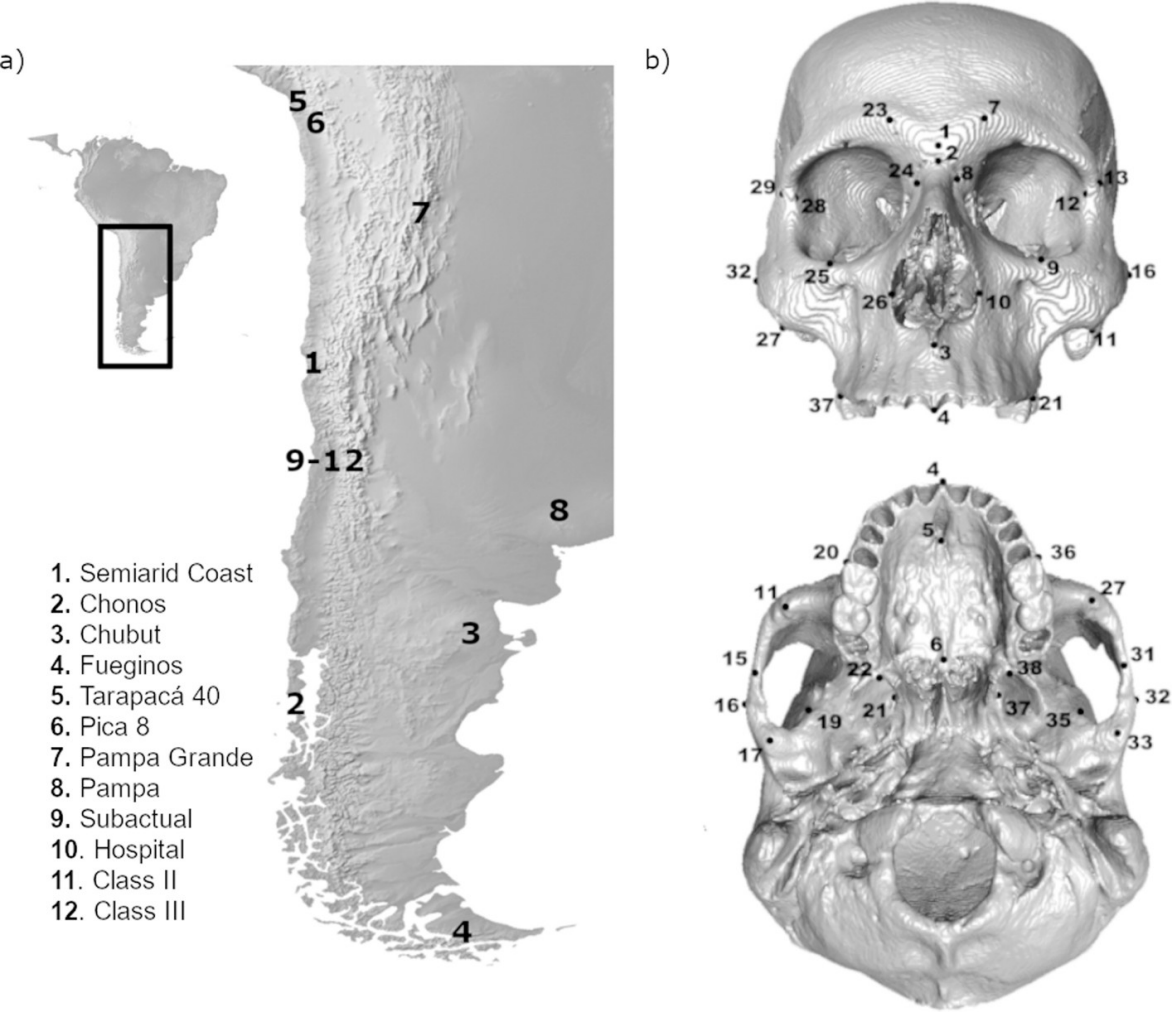
Geographical location of the sample and map of landmarks used. a) Geographical origin of the studied samples (map image obtained from http://www.naturalearthdata.com); b) Landmarks used in the study. See description in Table 2.

We used virtual high-resolution 3D models of 186 crania from pre-Hispanic and modern populations of Chile and Argentina (Table 1 and Fig 1A). The sample contrast in their geographic location, maxillomandibular occlusal relationship, chronology and subsistence economy. Individuals were classified in groups describing how masticatory loads were exerted during life: masticatory load intensity (according to their subsistence economy following the protocols outlined in previous studies [38–40, 42, 43]), and maxillomandibular relationship (which here describes the relative position of the maxilla and mandible in the sagittal plane and is used as a proxy for changes in masticatory load direction). This resulted in five groups in total: two for masticatory load intensity, two for maxillomandibular relationship and one for both factors. High Load (HL) and Intermediate Load (IL) are groups characterized by their masticatory load intensity and had a harmonic dental occlusion (i.e. without signs of anatomically extreme variants). Class II (CII) is classified as having mandibular retrognathia and equivalent to an extreme skeletal class II. Class III (CIII) is classified as having mandibular prognathism and is equivalent to an extreme clinical class III. Finally, Mild Load/Class I (ML/CI) corresponds to the control group for both factors; they are individuals who exerted mild loads during life and also had a harmonic dental occlusion.

**Table 1:** Sample description.

Group	Sample/Origin	Geographic region	N (F/M/?)
HL	Chonos	Southern Chile coast	0/0/2
	Semiarid Coast	Northern Chile	14/14/0
	Chubut	Patagonia, Argentina	4/10/0
	Fueguinos	Tierra del Fuego, Argentina	4/10/3
IL	Tarapacá 40	Northern Chile	6/6/2
	Pica 8	Northern Chile	11/6/2
	Pampa Grande	Northwestern Argentina	12/1/0
	Pampa	Patagonia, Argentina	9/9/0
ML/CI	Subactual*	Central Chile	5/7/0
	Hospital*	Central Chile	11/18/0
CII	Class II*	Central Chile	10/3/0
CIII	Class III*	Central Chile	2/6/0

HL = High Load; IL = Intermediate Load; ML/CI = Mild Load/Class I; CII = Class II; CIII = Class III; F = Female; M = Male; ? = indeterminate sex;. Groups with

* share the same origin (Santiago de Chile). For more details see S1 Table.

Craniofacial shape was compared using 3D landmark-based geometric morphometrics, an established technique that examines the geometrical properties of an object that are independent of their size, rotation and translation [44, 45]. These properties, namely shape variables, are the variables that are used in multivariate statistical analyses. Two sets of analysis were performed, a general one accounting for the face, and a more specific one, focusing in the inferior aspect of the maxilla (including the pterygoid process).

The HL group is composed of hunter-gathers, characterized by a subsistence practice with little or basic extraoral processing, resulting in tougher, mechanically challenging food items that require large and sustained occlusal forces [14, 46–48]. For this group we used four collections: Semiarid Coast, Chubut, Chonos, and Fueguinos. The Semiarid Coast sample (~4000–3000 BP, 4460–3120 cal BP) is a pool of individuals from three geographically and temporally similar sites from the coastal area of the semiarid north of Chile—La Herradura, Guanaqueros and Punta Teatinos—and who had a diet rich in marine resources [49, 50]. The Chonos sample (~1050-650BP, 1053–563 cal BP) comes from the Guaitecas Archipielago, in Southern Chile, with a diet characterized by marine resources (fish, shellfish and sea lion) [51, 52]. The Chubut sample (1500–900 BP) is from the Chubut Valley in the Argentinean Patagonia region [53]. These individuals had a mixed diet comprised of marine and terrestial resources, with a higher degree of the latter [54]. The Fueguinos correspond to a historic collection (post Spanish contact, without a known chronology) composed by Selk’nam and Yámanas individuals: the former had a more extensive terrestrial component in their diet, while the latter consumed more marine resources (shellfish, penguins, whales, etc.) [55].

The IL group is composed of horticulturists and agriculturalists, characterized by plant domestication, with a more sedentary lifestyle and knowledge of sowing and harvesting [56]. Food processing is also greater than the hunter-gathers groups, with grinding technology and preparation of alcoholic drinks (e.g. *chicha*) [56]. Their processing technologies led to the production of less mechanically challenging food items, decreasing the masticatory forces needed to chew them [57]. We used four samples for the IL groups: Tarapacá 40, Pica 8, Pampa Grande and Pampa. Tarapacá 40 is an pre-Hispanic cemetery located in northern Chile used during the formative period (3060–1410 cal BP), with an important consumption of C3 plants (e.g. Algarrobo or *Prosopis chilensis*) and little consumption of maize and marine resources [58]. The Pica 8 (1286–552 cal BP) sample was excavated in northern Chile, and was part of the Pica-Tarapacá complex dating to the Late Intermediate Period (900–1450 AD), located in close proximity to the Tarapacá 40 cemetery [59, 60]. Their diet was characterized by high consumption of maize and very scattered consumption of marine resources [58]. Pampa Grande is a group of the Candelarian period (400–650 AD) from the North-Western region of Argentina, with a diet based on wild and domesticated plants, such as Chañar (*Geoffroea decorticans*), beans and maize [61, 62]. The Pampa sample is a horticulturalist group that lived in the Argentinean Pampa of the Patagonia region roughly between the 17th and 18th centuries [63–66].

The ML/CI, CII and CIII groups are composed of urban individuals whose diet is characterized mainly by highly chemically and mechanically processed food [46]. The individuals in these three groups are from Santiago, Chile and differ in their maxillomandibular relationship. The ML/CI group includes one osteological and one living sample. The first comes from the Subactual Collection of Santiago de Chile (previously known as the *Cementerio General* Collection). This collection includes osteological material dating from 1960 to 1986, and in most cases with known names, sex, age, birth and death dates [67]. The remaining individuals of the ML/CI group correspond to patients from the teaching hospital of the University of Chile (*Hospital Clínico Universidad de Chile*). These individuals have a normal maxillomandibular relationship, with a harmonic occlusion in their permanent molars and an overjet (anteroposterior distance between upper and lower incisors) between 2 and 4 mm. The individuals in CII and CIII also come from the database of a medical facility in Santiago, Chile (*Instituto de Cirugía*, *Ortodoncia*, *Rehabilitación Oral y Maxilofacial*). The CII sample presents a severely retrognathic mandible, with an overjet over 5 mm. On the other hand, the CIII individuals have a profile equivalent to skeletal type class III, with a very prognathic mandible whose incisors are positioned anteriorly to the maxillary incisors.

All crania were selected according to their state of conservation preservation, including only those with complete craniofacial structures. The modern individuals and most of the archaeological sample had their associated mandibles, which were used only for the assessment of maxillomandibular relationship. When the mandible was not present, clinical parameters such as occlusal plane inclination, asymmetry and tooth axis inclination, as well as height within the arch were used to rule out severe alterations of the maxillomandibular relationship and dental extrusion due to absence of the antagonist tooth. Individuals with small signs of artificial cranial deformation—a widespread practice in the pre-Hispanic Andean world and present in some individuals from Pica 8, Tarapacá 40 and Pampa Grande [58, 62, 68]—were included because previous research has shown that this modification does not significantly affect the shape of the facial skeleton or its mechanical response during biting [69]. Age and sex for the archeological samples were estimated using cranial and postcranial (when available) features as described by Buikstra and Ubelaker [70]. Only adult individuals–with an ossified spheno-occipital synchondrosis and/or erupted third molars–were included in the analysis. There were individuals whose sex could not be determined. During previous work [71], shape variables (PC scores explaining up to 75% of the observed variance) of a part of the current sample was used as reference to perform a discriminant analysis to classify the undetermined individuals as female or male. Subsequently, sex was used as a dummy variable (female = 0, male = 1) in a regression of shape variables against sex to obtain the residuals (shape variables free of the effect of sex) for the subsequent analysis [72]. These analyses were conducted with the software PAST v.3.16 [73] and MorphoJ [74]. Three-dimensional surface files of most of the sample were created by threshold-based and manual segmentation of medical CTs using the software Avizo v.9.1 (Science Visualization Group, Burlington, USA). The only exception was the sample from the Semiarid Coast, whose surface files were generated during a previous study using a HD NextEngine laser surface scanner [50]. Thirty-eight craniofacial landmarks (Fig 1B and Table 2) were placed at glabella, the orbits, nasal aperture, maxilla, zygomatic and temporal regions with Avizo software. For the few individuals with missing landmarks due to bone damage, coordinates were estimated using the estimate.missing function (based on the thin-plate spline function) of the package Geomorph in R (www.r-project.org). Since the cranium reflects a small degree of asymmetry that is not of interest in this study, only the symmetric component of shape variation was used for all morphometric analyses. The effect of intraobserver error was assessed by double digitizing 20 individuals with the results indicating a negligible intraobserver error (S2 Table). That is, interindividual variation is significantly larger than intraindividual variation derived from repeated landmarking in a Procrustes ANOVA [See example in 26]. The level for statistical significance was set to 0.05.

**Table 2:** Definition of the landmarks used.

n°	Landmark	Definition
**1**	Glabella	Most prominent midline point located above the frontonasal suture.
**2**	Nasion	Midline point of the intersection between the frontonasal and internasal sutures
**3**	Point A†	Most concave point between the anterior nasal spine and Prosthion at the midplane.
**4**	Prosthion†	Most anterior point on the midline on the alveolar process of the maxilla
**5**	Foramen incisivum†	Point where the medial palatal suture meets the posterior margin of the foramen incisivum.
**6**	Staphylion†	Most posterior point on the interpalatal suture.
**7, 23**	Supraorbital torus	Most anterior point of supraorbital ridge.
**8, 24**	Maxillo-frontale	Point where the anterior lacrimal crest of the maxilla meets the frontomaxillary suture.
**9, 25**	Zygoorbitales	Point where the orbital rim intersects the zygomaticomaxillary suture.
**10, 26**	Alare	Most lateral part of the nasal aperture in a transverse plane.
**11, 27**	Zygo-maxillare †	Most inferior point of the zygomatico-maxillary junction.
**12, 28**	Frontomalare orbitale	Point where the frontozygomatic suture crosses the inner orbital rim.
**13, 29**	Fronto-zygomatic	Most lateral point of the fronto-zygomatic junction.
**14, 30**	Fronto-temporal angle	Point at the intersection between frontal and temporal processes of the zygomatic bone.
**15, 31**	Zygo-temporal inferior†	Most inferior point of the zygomatic-maxillary junction.
**16, 32**	Zygomatic arch medial	Most lateral point of the zygomatic arch.
**17, 33**	Articular tubercle	Lowest point of the articular tubercle
**18, 34**	Zygomatic root posterior	Most posterior-superior point of the intersection between the zygomatic root and the squama of the temporal bone.
**19, 35**	Zygomatic root anterior	Most anterior point of the intersection between the zygomatic root and the squama of the temporal bone.
**20, 36**	First molar†	Most buccal and mesial point of the junction of the M1 and the alveolar process. If M1 is absent, the landmark is in the lowest most buccal point of the interalveolar septum between the second premolar and the next molar.
**21, 37**	Superior pterygoid origin†	Most superior point of the origin of the medial pterygoid muscle.
**22, 38**	Inferior pterygoid origin†	Most inferior point of origin of the medial pterygoid muscle.

† Landmarks used in the analysis of the inferior aspect of the maxilla.

General shape variation of the pooled sample was examined using principal components analysis (PCA) of shape variables in MorphoJ. In order to assess whether there was support for our prediction of a stronger morphological response of the inferior aspect of the maxilla (named simply “maxilla” hereafter), a PCA focused on only the maxillary landmarks was also performed. The allometric effect on general craniofacial shape variation was assessed by regressing shape variables against centroid size in MorphoJ and the results are discussed regarding its relationship to the studied factors.

A canonical variate analysis (CVA) was performed to statistically test the hypothesis of shape differences (craniofacial skeleton and isolated maxilla) among groups due to due to masticatory load intensity and maxillomandibular relationship. Due to the large number of dependent shape variables generated by our sample, CVA statistical results were corroborated using permutation-based MANOVA (PERMANOVA) [75] on the PC scores, explaining up to 80% of the variance for each analysis. To visualize said shape changes, hypothetical shapes depicting the most characteristic features of the load intensity and maxillomandibular relationship groups were created in Avizo based on the extremes scores of the CVA for each.

We also assessed whether the expected differential response of the craniofacial skeleton and the maxilla may be due to a structural “disconnect” (i.e. a reduced degree of integration) between cranial parts: the upper portion of the face (including glabella on the frontal bone), related indirectly to load bearing functions, and the directly loaded maxilla. This is based on the idea suggested by Toro-Ibacache, Zapata Muñoz [30] of an ontogenetic mechanism that regulates, through the maxilla, how masticatory forces affect the rest of the cranium. The strength of integration within groups was assessed using the PLS correlation coefficient (r_PLS_), based on a common Procrustes fit for the entire 38 landmark configuration and its statistical significance tested with 1000 permutation rounds. To assess whether load bearing can affect the degree of integration between the upper face and the maxillary aspect, we compared the magnitude (or “strength”) of integration among individuals within the two sets of groups using the method by Adams and Collyer [76]. This method uses z-scores as a measure of integration strength; which are based on the normalized r_PLS_ values that eliminates the confounding effect of different numbers of individuals within each group. The analyses were performed using the functions two.b.pls and compare.pls in the Geomorph package in R, respectively. Visualization of the covariation patterns within groups was made using within-configuration partial least square analysis (PLS) in MorphoJ and Avizo.

This study has been approved by the Ethical Committee of *Facultad de Medicina*, *Universidad de Chile* (CEISH, N° 203–2015). It was approved based on the Chilean law for scientific research with human material and comprises: 1) how the sample was obtained (from museum collections and databases, and from anonymized retrospective databases provided by the Hospital/Clinic without the need of individual’s consent); and 2) how the sample is used during research. The morphometric data of the sample is provided as supporting information (see S3 Table). Note that the use of these data for another research project needs to be authorized by the corresponding institutions housing the original skulls and/or CT data (see S1 Table).

## Results

**Fig 2:**
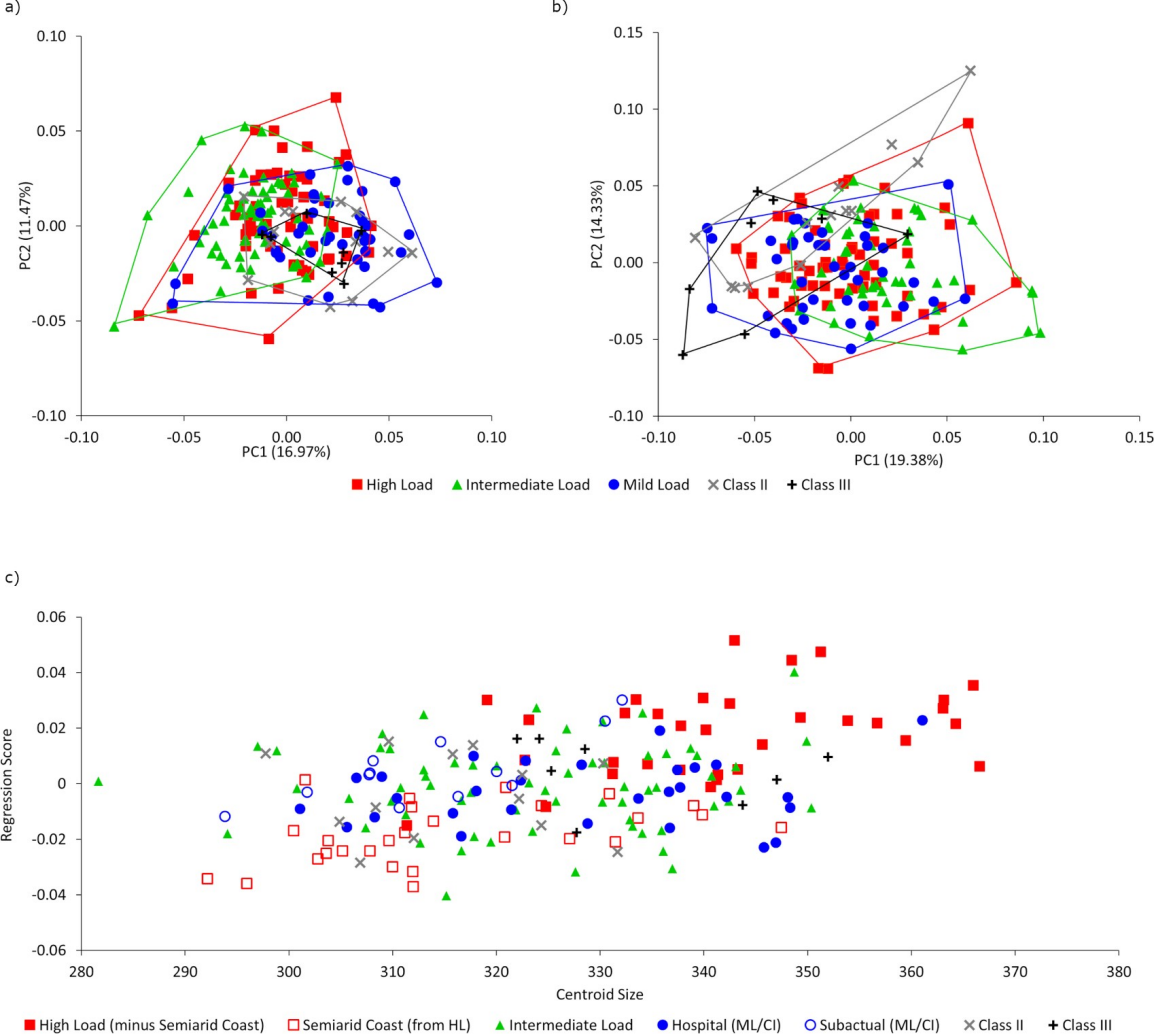
Principal component analysis and allometry assessment. a) PCA of the shape variables of the craniofacial skeleton showing the first and second components (PC1 and PC2). Values in parentheses correspond to the proportion of variance explained; b) PCA of the inferior aspect of the maxillary showing the first and second components (PC1 and PC2). Values in parentheses correspond to the proportion of total variance explained; and c) Regression of craniofacial shape variables on centroid size.

In the PCA of the pooled sample, for the upper face and maxilla landmark configuration (Fig 2A), the two first principal components (PC1 and PC2) show large areas of overlap on both axes and no separation among groups. The ML/CI group shows the broader range of variation along PC1, overlapping extensively with all the groups and completely overlapping the two altered occlusion groups.

Fig 2B shows results of the exploratory PCA of the maxilla, which indicates extensive group overlap on the two first PCs. While there is overlap among all groups on both axes, the CIII individuals tend be slightly clustered towards the negative end of PC1, while CII is distributed across PCs 1 and 2, giving it a slightly more distinctive pattern of variation than the other groups.

Similar to the PCA, the regression of shape against centroid size (Fig 2C) shows overlap among all groups, meaning that allometry (which is statistically significant; % of total variation explained = 1.61; p-value = 5x10^-4^) depicts features that are shared by all individuals, irrespective of their load intensity or maxillomandibular relationship. Within groups, some individuals have a somewhat more distinctive pattern, skewed towards either the smallest or largest centroid sizes (subgroups “Semiarid Coast” and “Subactual”, in particular).

The CVA (Table 3), shows statistically significant differences in Procrustes distances between all masticatory load intensity groups for the craniofacial and maxilla analyses, even after Bonferroni correction. The differences due to maxillomandibular relationship are less marked; before Bonferroni correction, and differences between most skeletal classes are statistically significant, with the exception of pairwise distances between CII and CIII in the craniofacial analysis. After the Bonferroni correction the only statistically significant difference remain in the analysis of the isolated maxilla between ML/CI and CII, and ML/CI and CIII. The pairwise PERMANOVA analysis based on PCs explaining up to 80% of the observed variance (PCs 1 to 16 and 1 to 13 for the craniofacial structure, per load intensity and maxillomandibular relationship respectively; and PCs 1 to 7 and 1 to 8 for the isolated maxilla per load intensity and maxillomandibular relationship) corroborated the CVA results shown in Table 3.

**Table 3:** Procrustes distances of the CVAs and PERMANOVA.

		Masticatory Load Intensity				Maxillomandibular relationship			
			HL	IL	ML/CI		ML/CI	CII	CIII
**Procrustes CVA**	**Craniofacial**	HL		**< .0001**	**< .0001**	ML/CI		0.0392*	0.0231*
		IL	0.0239		**< .0001**	CII	0.0269		0.3196
		ML/CI	0.0438	0.0359		CIII	0.0332	0.0303	
	**Maxilla**	HL		**< .0001**	**< .0001**	ML/CI		**0.0018**	**0.0031**
		IL	0.0303		**< .0001**	CII	0.0443		0.0379*
		ML/CI	0.0415	0.0501		CIII	0.0501	0.058	
**PERMANOVA**	**Craniofacial**	HL		**0.0003**	**0.0003**	ML/CI		0.1182	0.0654
		IL	**0.0001**		**0.0003**	CII	**0.0394**		1
		ML/CI	**0.0001**	**0.0001**		CIII	**0.0218**	0.3638	
	**Maxilla**	HL		**0,0003**	**0.0003**	ML/CI		**0.0051**	**0.0138**
		IL	**0.0001**		**0.0003**	CII	0.0017		0.1131
		ML/CI	**0.0001**	**0.0001**		CIII	0.0046	0.0377	

Procrustes CVA: The Procrustes distances (lower diagonal), and their associated p-value (upper diagonal). Significant values are showed in bold and those non-significant after Bonferroni correction are marked with an

*; PERMANOVA: Matrix of pairwise differences of PERMANOVAs (based on PC scores) in the lower diagonal, their associated p-value and in the upper diagonal, and p-value after the Bonferroni correction. HL = High Load; IL = Intermediate Load; ML/CI = Mild Load/Class I; CII = Class II; CIII = Class III.

**Fig 3:**
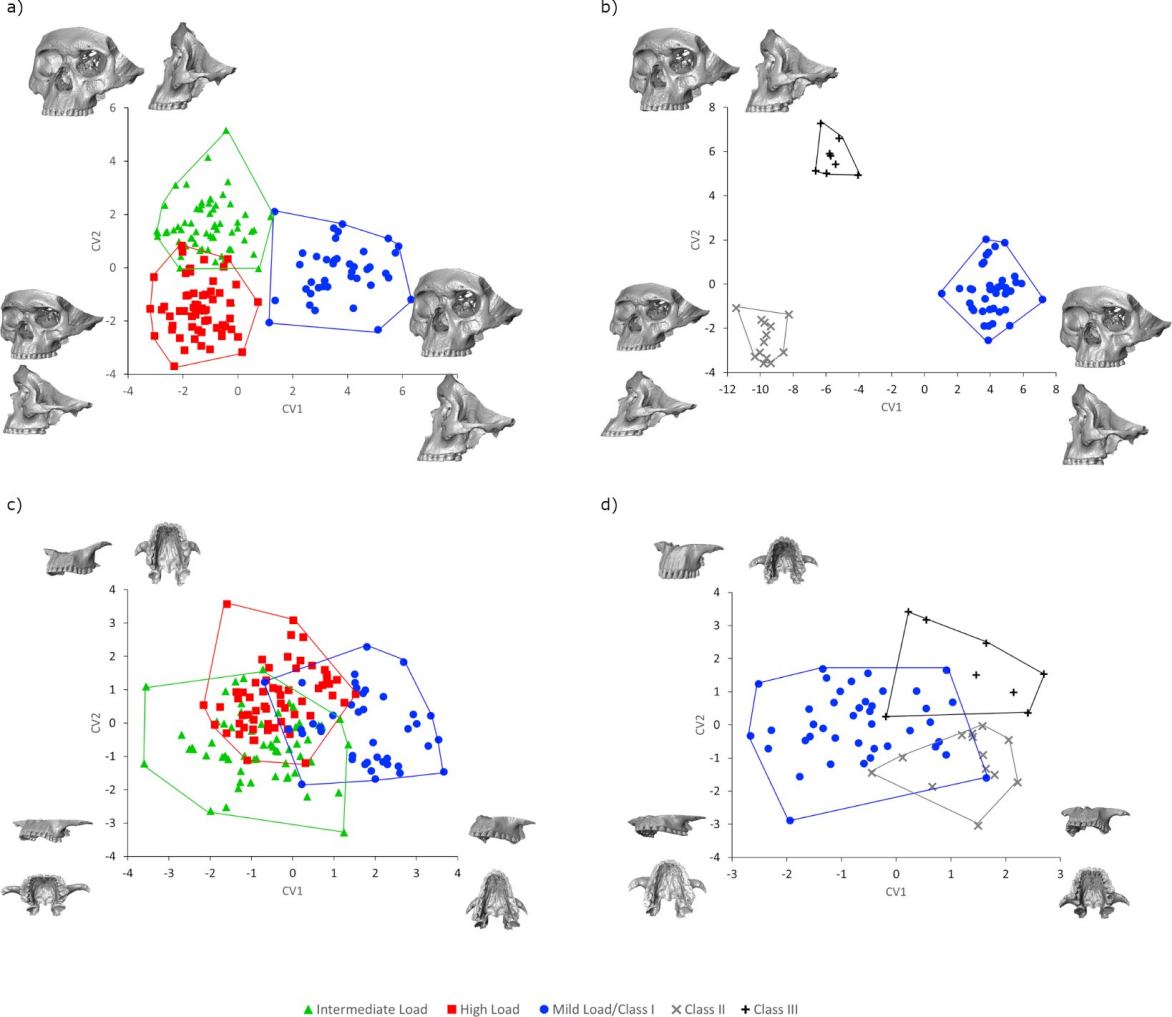
Canonical variance variate analysis of the craniofacial and maxilla, with their hyphotetical shapes of their extremes. a) Craniofacial CVA of masticatory load intensity; b) Craniofacial CVA of maxillomandibular relationship; c) Maxillary CVA of load intensity; and d) Maxillary CVA of maxillomandibular relationship. The warpings for all the analysis have been magnified 3 times.

Fig 3A displays the CVA and craniofacial shape differences by masticatory load intensity and maxillomandibular relationship. For masticatory load intensity, HL and IL form clusters with small areas of overlap. The first axis (CV1) depicts main differences in craniofacial inclination with the modern, ML/CI individuals showing more anteroposteriorly inclined profiles, while the zygomatics and maxilla are more anteriorly placed with respect to nasion. The opposite is shown by HL individuals, with a more vertically oriented and less elongated profile. IL individuals, at the negative end of CV1 and positive end of CV2, distinguish themselves by an anteroposteriorly shortened craniofacial skeleton and a vertically inclined profile. Both IL and ML/CI share rounded, vertically elongated faces with respect to HL. In the CVA for maxillomandibular relationship (Fig 3B) there is distinct separation between groups. The most distinctive group is CII which, as predicted, has a marked anteroposteriorly inclined profile and a shortened alveolar process. ML/CI and CIII, at the positive ends of CV1 and CV2, share a more vertically oriented profile (slightly more vertical in ML/CI), with ML/CI also depicting a slightly less anteroposteriorly elongated face.

For the CVA of the maxilla among masticatory load intensity groups (Fig 3C), CV 1 depicts anteroposterior variation, with ML/CI showing a more elongated and narrow maxilla (for the alveolar process and palate) when compared to IL and HL. CV2 shows variation in the vertical and horizontal extension of the maxilla, with HL having a taller and broader maxilla and palate in all directions. For the maxillomandibular relationship (Fig 3D) CV1 depicts horizontal variations of the maxilla, with mostly CII and CIII individuals showing an anteroposteriorly shorter maxilla, which is also more anteriorly placed with respect to the zygomaticomaxillary suture. CV2 corresponds to vertical extension of the maxilla, with CIII showing a taller maxilla and a deeper palate.

**Fig 4:**
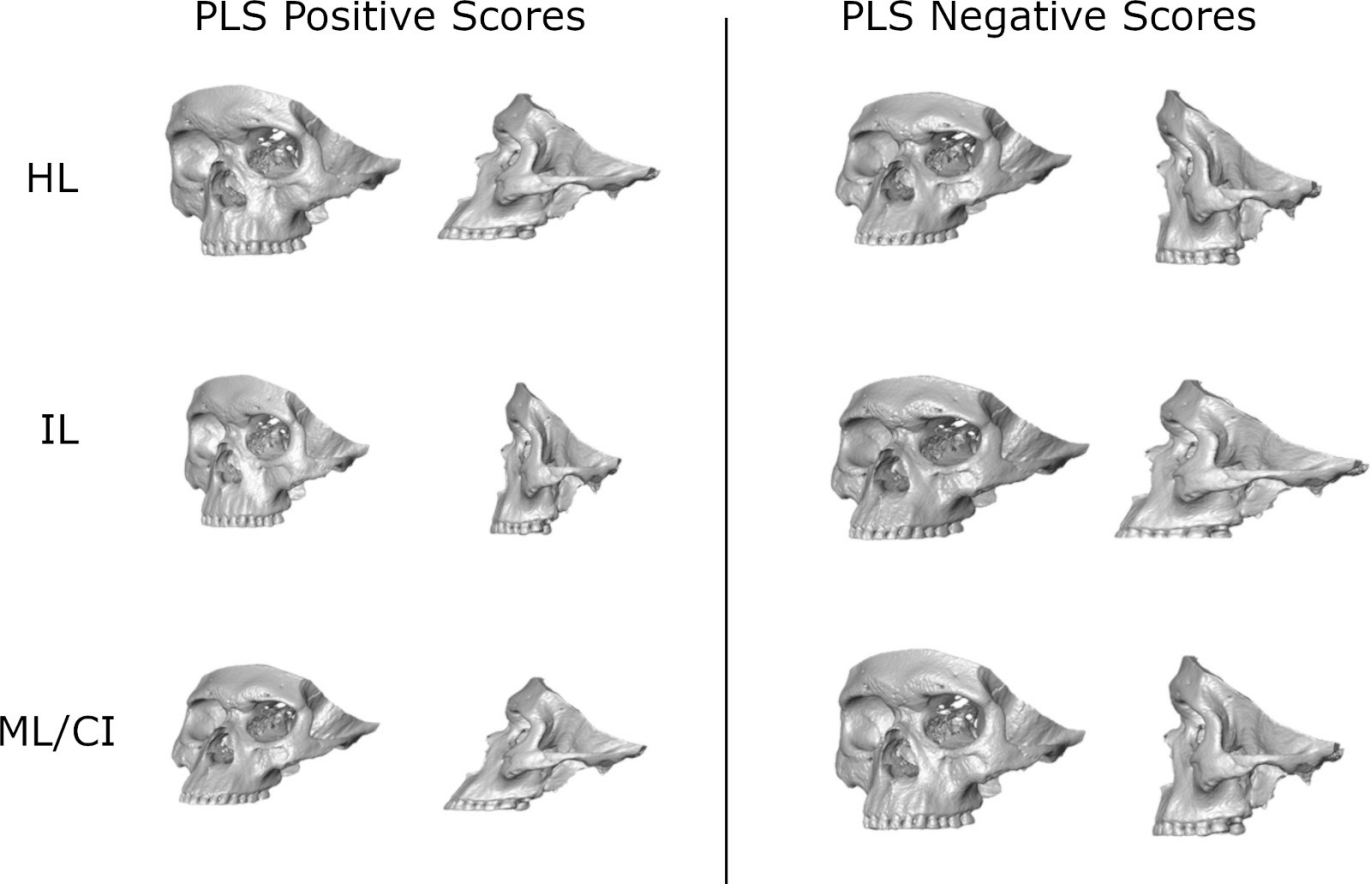
Visualization of the covariation patterns of the statistically significant within-configuration partial least square analysis. Magnified 3 times.

The assessment of morphological integration between the upper portion of the face (including the lower part of the frontal bone) and the maxilla based on the within-groups PLS analysis showed that all groups, except for CIII (and CII after Bonferroni correction), have a statistically significant degree of morphological integration between the upper face and the maxilla (Table 4). Comparatively, however, z-scores for HL and IL show the highest values, indicating that these groups have a greater degree of integration between the two regions than the other three groups. This degree of integration decreases gradually from ML/CI to CII and CIII. Also evident in Table 4 are differences in the degree of integration, with the largest between CIII and the archaeological samples, HL and IL. However, the comparison of pairs within the load intensity groups showed no statistically significant differences after the Bonferroni correction. Among individuals with different maxillomandibular relationships, only CIII and CI show a statistically significant difference. Fig 4 depicts the patterns of shape covariation within each group (magnified three times), except for CII and CIII, because they represent non-significant degrees of integration between the upper face and the maxilla. The extreme values of the first PLS axis are shown for each group. In general terms, all groups show similar patterns (but not degree) of covariation between the upper face and the maxilla that results in either a more inclined or vertical profile, with small variations that fall within the features described above in the CVA.

**Table 4:** Degree of morphological integration between the upper face and the maxilla within and between groups.

Degree of morphological integration (pairwise)						Degree of morphological integration (within groups)		
	HL	IL	ML/CI	CII	CIII	r_PLS_	P	z
HL		0.414	0.0390*	**0.001**	**< .0001**	0.865	**0.001**	6.571
IL	0.217		0.0239*	**< .0001**	**< .0001**	0.907	**0.001**	6.976
ML/CI	1.759	1.979		0.103	**< .0001**	0.785	**0.001**	3.654
CII	3.015	3.235	1.264		0.057	0.891	0.038*	1.701
CIII	5.299	5.589	3.107	1.589		0.862	0.645	-0.358

The matrix of pairwise differences shows PLS effect size (lower diagonal), and their associated p-value (upper diagonal). For within-group integration rPLS and their respective p-values and z-scores are shown. Statistically significant values are shown in bold and those non-significant values after Bonferroni correction are marked with an asterisk; HL = High Load; IL = Intermediate Load; ML/CI = Mild Load/Class I; CII = Class II; CIII = Class III.

## Discussion

The present study investigates how masticatory load intensity and maxillomandibular relationship influence shape variation of the human skull. Using a sample of archaeological and modern/urban populations from the American Southern Cone, we tested the hypothesis that there is a relationship between masticatory load intensity, maxillomandibular relationship and craniofacial shape, which characterized their patterns of variation.

We chose a methodological approach to classify masticatory load intensity based on subsistence economy similar to those used in previous research [38, 39, 42, 43]. This approach has generated debate among researchers, and the classification probably omits some details about cultural variation among the groups (e.g., not all the hunter-gathers ate exactly the same foods at a given time and they may have processed food differently). However, the extent to which archaeological data can describe the exact nature of dietary choices over time and space (and perhaps more importantly, mode of consumption) among prehistoric groups is difficult to ascertain, and hence this relationship between general subsistence economy and masticatory load serves as a proxy. The classification of maxillomandibular relationship was made using clinical criteria on extreme cases of alterations; thus, strict classifications based on cephalometric references were avoided. Based on our results, there is weak support for the main hypothesis of this study, but greater support for our predictions on the maxilla, which are discussed below.

The PCA for the craniofacial skeleton (Fig 2A) displays overlap among all the groups, without any visible group distinction based on masticatory load intensity or maxillomandibular relationship. In the allometry analysis there is also an overlap between the groups irrespective of their load intensity and maxillomandibular relationship. HL shows a greater degree of variation and the largest centroid sizes. Regarding this greater degree of variation along the regression line, a more detailed review of these data show that when HL is subdivided in groups according to their origin, these are not distributed homogeneously in the allometry data cloud. Instead, individuals from Southern Chile and Argentina are considerably larger (and subsequently, of different shape) than those from Northern Chile (Fig 2C, red squares vs. open red squares). Modern individuals, having the same origin regardless of their maxillomandibular relationship, were distributed more homogeneously across centroid sizes. The same occurs with IL. Thus, allometry could be a more important factor in determining shape variation. The large variation in the modern sample, irrespective of their origin or maxillomandibular relationship, could be explained by different factors, but perhaps more importantly, by the lack of one of them: large masticatory forces. After analyzing the external morphology and the amount and distribution of cortical bone in mandibles of individuals with differences in load intensity and maxillomandibular relationship, Toro-Ibacache, Ugarte (19) suggested that the lack of masticatory load reduced the functional constraints on skull development, giving way to a greater range of morphological variation that accounts for the action of different factors, which can be both environmental and genetic.

This poses an interesting question regarding the use of craniofacial traits to reconstruct the history of prehistoric and/or historic populations, particularly those where the masticatory constraints are reduced, such as the IL and ML/CI groups. This issue becomes more evident since the group with HL (except the group from the Semiarid Coast) tends to cluster distinctively in the allometry analysis (Fig 2C), perhaps indicating a shared functional environment, and even maybe a basal susceptibility different to that of the Semiarid Coastal individuals, which results in morphological similarities and a lower degree of variation with respect to size. Within these functional factors, other factors such as climatic adaptations, cannot be disregarded. Indeed, the northern Semiarid Coast individuals show some traits that differentiate them within the HL group (data not shown). It has been demonstrated that individuals from extreme environments (in this case, the Southern hunter-gatherers) tend to have larger crania [7, 77], which agrees with the distribution in our allometry analysis. Thus, high masticatory load plus extreme climatic conditions could be canalizing skull development, resulting in a relatively lower range of morphological variation when compared to the individuals that developed under more favorable climatic conditions or who ate less mechanically demanding diets.

Using a more detailed plot for the individuals without extreme functional constraints, our results showed that the Subactual group was smaller when compared to the Hospital sample. (i.e. many of them trend toward smaller sizes in the allometry analysis, Fig 2C, open blue circles vs. blue circles). Although both belong to the ML/CI group and were consumers of highly processed food, it is plausible that several of the Subactual individuals are smaller due to the nutrition deficiency previously reported among them [67]. Indeed, the link between bone morphology and nutrition has been suggested previously [19, 34, 78], and in this study, it could explain the range of morphological variation and trend towards smaller phenotypes.

The CVA (Fig 3A and 3B) showed limited influence of masticatory load (by intensity and maxillomandibular relationship) on facial shape, indicating weak support for our hypothesis. Instead, the CVA of face shape by masticatory load intensity showed more distinguishable differences between the archeological (HL and IL) and the ML/CI groups. Differences among masticatory load intensity groups could be due to load intensity, but when considering the results of the maxilla this is less likely. Other external factors, such as those discussed above could also be the cause, but the contribution of ancestry should not be disregarded. Morphological traits shared by the archeological samples and the modern ML/CI group could be attributed to individuals of Native American and European ancestry, which is to be expected when the archeological samples are Amerindian and the modern samples are contemporary urban Chilean individuals, many of whom are of mixed European and Native American descent [67, 79]. For the CVA of craniofacial shape by maxillomandibular relationship (Fig 3B), there is a lower range of within-group variation among ML/CI, CII and CIII, resulting in more marked differences. The striking differences among individuals with different maxillomandibular relationships may be due to the presence of more “extreme”, morphological characteristics within each group, particularly regarding the inclination of their profile (Fig 3B). These differences are to be expected since clinically the faces of these individuals have characteristic profiles. The basis for these distinctive features could be linked to genetic factors, which can be enhanced by the lack of strong masticatory forces and their altered directions [32].

Interestingly though, in the case of the maxilla (Fig 3C and 3D) which is directly loaded with varying force intensity and directions, there are no distinctive features for each group, as we predicted. Instead, there is comparatively more overlap among groups of masticatory load intensity (Fig 3C) and maxillomandibular relationship (Fig 3D). However, the represented features of the maxilla vary to a larger degree, and these degrees of variation are shared among groups. Masticatory load intensity (Fig 3C) continues to distinguish the modern group (ML/CI), but the groups are less distinctive among ML/CI, CII and CIII (Fig 3D), who share load intensity but vary in their force direction. These results suggest that masticatory loads do affect the shape of the maxilla to a greater degree but in a very heterogeneous, non-deterministic way. That is, the maxilla can plastically change in response to small variations in masticatory forces, while the craniofacial skeleton as a whole and particularly the upper portion of the face is more constrained. Taken together, differences between the face and maxilla support the idea that these relate differently to masticatory load (in both intensity and direction), in agreement with von Cramon-Taubadel [38] and Noback and Harvati [80].

Beyond the confirmation of a different effect of force on the face and isolated maxilla, it is of interest to propose mechanisms by which this may occur. In this regard the results of the PLS were very informative. In the case of CII, CIII and, to a lesser extent, ML/CI, there is a lower degree of integration between the upper face and the maxilla than HL and IL (Table 4). That is, they are relatively disconnected (or modular) in the way they covary due to their different functional environment. HL and IL, who would during their lives have exerted higher magnitudes of masticatory forces, have a higher z-score (Table 4), than the modern groups (ML/CI, CII and CIII). This could suggest that when masticatory forces are of a certain magnitude, they would strain and shape the maxilla in such a way that the maxilla would need to “drag” the upper face in the adopted shape, increasing the integration level between the parts. Conversely, when masticatory load intensity is reduced, as it happens due to the highly procced food-diet of the modern groups, the maxilla does not constrain the development of the upper face in response to other functions (breathing, nervous system support), leading to diminished integration between the masticatory maxilla and the upper face.

These results partly differ with those reported by Spassov, Toro-Ibacache [26] in mice fed with soft and hard diet with or without developing a muscle dystrophy [26]. Spassov and colleagues demonstrated that mice with muscle dystrophy fed with soft diet -which results in a low force input of the developing skull- showed a higher integration between the neurocranium and the face. The analyzed parts were not exactly equivalent (cranial base and vault were analyzed in the mice) and thus differences between both results can be due purely to methodological choice and of course, to the differences in skull shape between species. However, it can be hypothesized that different species may show different degrees of integration under what can be assumed as “similar functional conditions”. This could be due to the fact that among very different species these functional conditions will always differ; brains, eyes and nasal cavities and their functions differ, and masticatory movements differ. Within humans though, our explanation to a force magnitude-dependent degree of integration is supported by a previous study by Toro‐Ibacache and O'Higgins [81]. This study showed that by using finite element analysis, masticatory forces first strain the maxilla and that when the magnitude increases, strains reach higher values in the upper portion of the face (including the lower part of the forehead). Bone strains are key in bone modeling and remodeling and thus, help in determining bone shape. It is important to highlight that even when there is a reduction of the integration between face parts, the growth and development of the structures still occurs within a certain range of variation, which is functional and that should respect the architecture of the face [82].

Based on the results of this study, masticatory load, in both intensity and maxillomandibular relationship, has a relatively weak influence on determining characteristics of craniofacial shape. Instead, masticatory load (intensity more than direction) would be important in determining the degree of constraint acting on developing bones and hence, the degree of morphological variation. The constraint reduces as masticatory load intensity does, increasing the degree of variation in individuals that are now under the relatively stronger effect of other variables, both genetic and environmental. Since other non-random factors–such as climate or altitude adaptation–have been found to have limited effects on craniofacial shape [7, 83], we adhere to the idea that neutral or random factors, such as population history or genetic drift [84] may explain craniofacial variation to a large degree. Although we recognize the utility of morphological variation as a proxy to evaluate population relationships [77, 83], we should not exclude the potential effects of environment, particularly in the absence of functional developmental constraints. For the inferior part of the maxilla, the influence of masticatory load intensity and maxillomandibular relationship is more apparent, especially in the case of individuals without constraining masticatory forces, like modern populations. Because of the role of the maxilla in the mastication process, we expected this region to reflect greater masticatory load than the rest of the face, but instead we see in it the larger degree of variation, with supports its role as a mediator or buffer between the forces of mastication and the upper structures of the face.

## Supporting information

S1 Table Sample information. Detailed information of the samples used in this study and the contact information to request for access.
(XLSX)

S2 Table Procrustes ANOVA of intraobserver error (repeated landmarking). Statistically significant p-values are shown in bold.
(XLSX)

S3 Table Morphometric data. Morphometric data used in the study.
(XLSX)
